# A Case of Transverse Myelitis Secondary to COVID-19 Infection

**DOI:** 10.7759/cureus.32297

**Published:** 2022-12-07

**Authors:** Rahul Borra, Neal T Patel, Raheel Shaikh, Mayur S Parmar, Sujatha Borra

**Affiliations:** 1 Medicine, Nova Southeastern University Dr. Kiran C. Patel College of Osteopathic Medicine, Fort Lauderdale, USA; 2 Foundational Sciences, Nova Southeastern University Dr. Kiran C. Patel College of Osteopathic Medicine, Clearwater, USA; 3 Neurology, AdventHealth Tampa, Tampa, USA

**Keywords:** treatment, mri, acute transverse myelitis, covid-19, sars-cov-2

## Abstract

Infection with COVID-19 (SARS-CoV-2) is associated with a variety of generalized and specific symptoms, including neurological complications of both the peripheral and central nervous systems. In this case report, we present the case of a previously healthy 55-year-old woman who was diagnosed with transverse myelitis following a previous infection with COVID-19. MRI showed progressive demyelination of the cervical and thoracic spinal cord, and cerebrospinal fluid (CSF) showed increased levels of protein and red blood cells and no markers of infection, including negative polymerase chain reaction (PCR) for COVID-19 antibodies. The patient was treated with a course of methylprednisolone, multiple treatments of plasmapheresis, and ongoing treatment with rituximab, all of which were well-tolerated. She was instructed to follow up as an outpatient with the neurologist and primary care physician five to seven days after hospital discharge.

## Introduction

COVID-19 is an infectious disease caused by the SARS-CoV-2 that emerged in Wuhan, China, in December 2019. It was declared a global pandemic on March 11, 2020. While infecting more than 629 million people worldwide and causing over 6.5 million deaths worldwide, COVID-19 has been noted to produce various symptoms throughout several organ systems. While COVID-19 primarily affects the pulmonary system, other systems such as the cardiovascular, renal, gastrointestinal, and nervous systems have been susceptible to the virus [1-3]. Some noted neurological symptoms include nonspecific symptoms such as headache, anosmia, and dizziness. In contrast, more severe neurological symptoms include cerebral thrombosis, delirium, stroke, encephalitis, ataxia, and conditions of neuronal demyelination, including transverse myelitis (TM) [3,4].

SARS-CoV-2 most commonly enters the body through inhalation of aerosolized respiratory droplets, which then attach to angiotensin-converting enzyme 2 (ACE2) receptors in the alveoli epithelium. However, ACE2 receptors have also been found in neurological cells such as neurons and glial cells [2]. The virus may enter through the olfactory bulb, which then enters the central nervous system, causing inflammation and damage, leading to neurological complications [4].

As TM is a devastating disease associated with considerable morbidity and mortality, recognizing the neurological sequelae of the disease is of utmost importance in guiding patient care. There are numerous proposed etiologies for TM, including paraneoplastic, autoimmune (perhaps due to multiple sclerosis or acute disseminating encephalomyelitis), and para-infectious causes (due to Herpes simplex virus or Mycoplasma pneumoniae) in which the body's response to the foreign pathogen mistakenly affects proteins in myelin. A few cases have been published regarding TM and COVID-19 as the complication is quite rare [5-10]. Here, we present the case of a healthy, immunocompetent woman who developed a case of TM after being infected with the COVID-19 virus.

Note: This paper was previously posted to the preprint server available at Research Square (https://doi.org/10.21203/rs.3.rs-1543376/v1) on July 8, 2022.

## Case presentation

A 55-year-old Asian woman with no notable family history, travel history, or past medical history presented to the emergency room with a primary complaint of generalized weakness, urinary incontinence, and tingling of the abdomen. The patient stated that she experienced these symptoms for approximately the past two weeks. In the initial presentation, she stated that she had previously experienced mild flu-like symptoms during the previous weeks but denied any symptoms at present. Due to hospital protocols at the time of admission, reverse transcription-polymerase chain reaction (RT-PCR) was used to test the patient for COVID-19 infection, and it was positive. It was suspected that the patient had a previous COVID-19 infection, which accounts for her positive result at the hospital. In the hospital, she was placed in isolation while further testing and imaging were ordered to determine the cause of her symptoms. A physical exam revealed 3+ hyperreflexia of the bilateral patellar and Achilles reflexes and a positive Babinski reflex bilaterally. Pinprick sensation revealed an almost complete loss of sensation to pinprick at the level of T5 dermatome and below bilaterally. There were no other pertinent positive findings on the initial physical examination.

**Figure 1:**
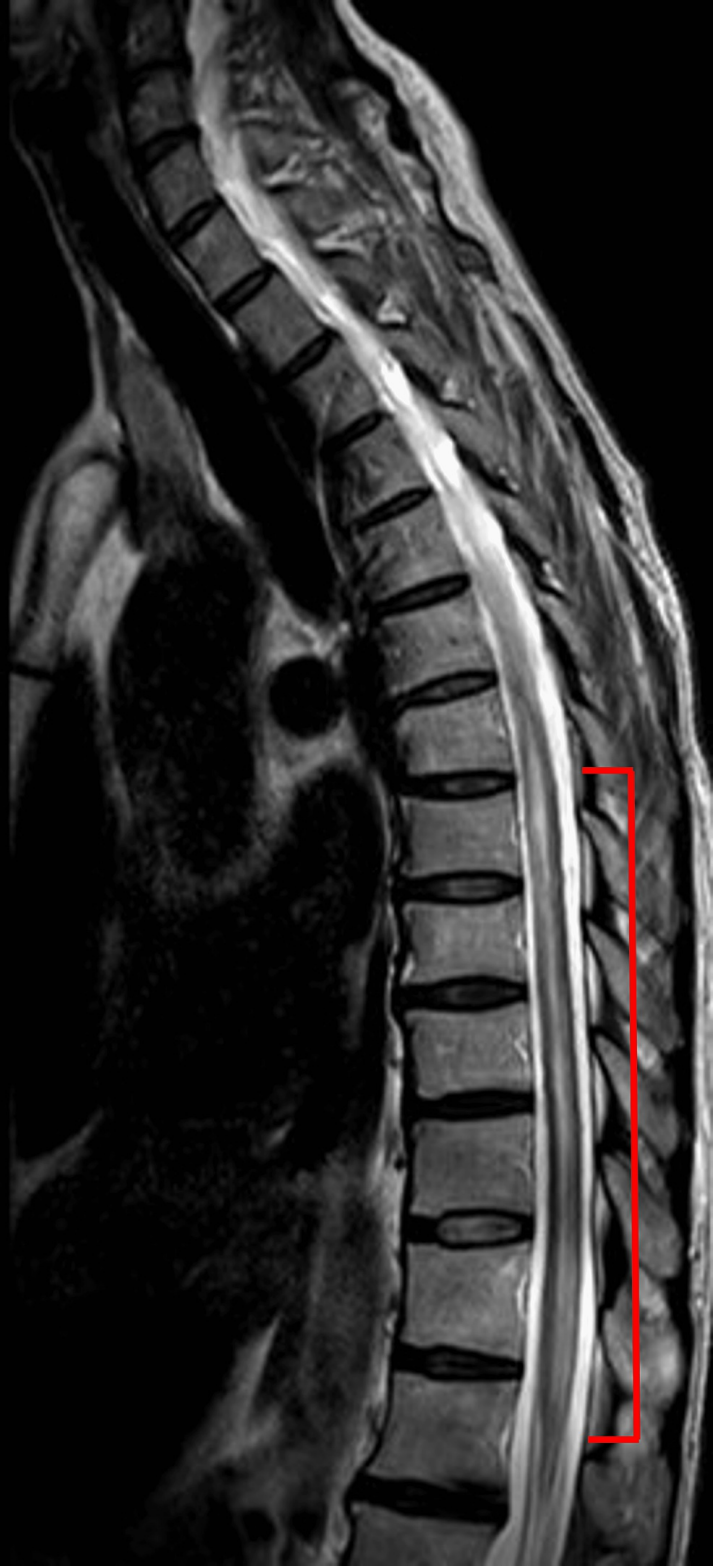
Hyperintensity at the level of C5-C6 extending down to the conus medullaris.

**Figure 2:**
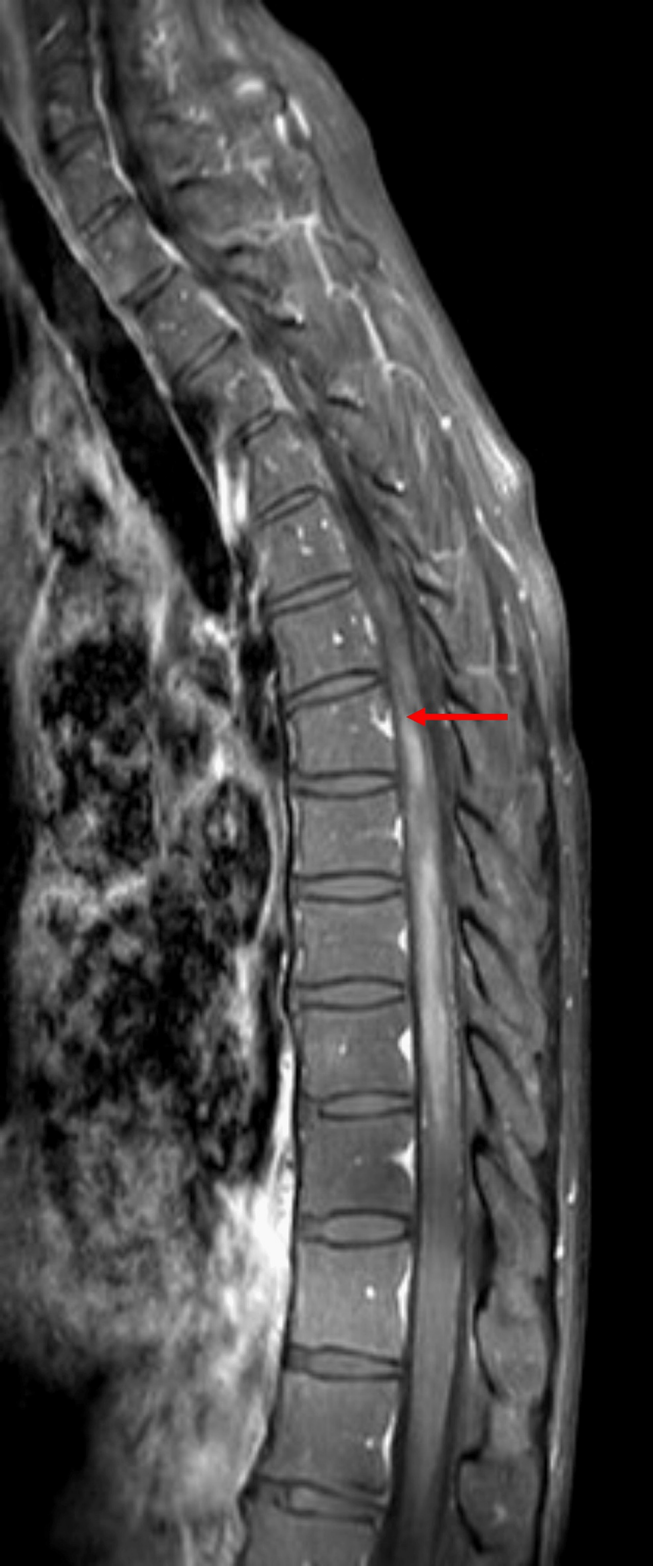
A 2-mm-long syrinx from about the levels of T7-T9.

Based on her initial presenting symptoms, an MRI of the cervical, thoracic, and lumbar spine with and without contrast was ordered, along with an MRI of the orbits with and without contrast and a CT scan of the brain without contrast. The MRI of the spine revealed hyperintensity beginning at the lower cervical spine at the levels of C5-C6, extending down to the conus medullaris (Figure 1). Along with this, a small syrinx approximately 2 mm in diameter was found extending from the levels of T7-T8 to T8-T9 (Figure 2). Along with these findings, the MRI report revealed disc osteophytes at C4-C5 and C5-C6. Bilateral pulmonary infiltrates were also found, and a follow-up chest radiograph was ordered. The MRI report of the orbits revealed a few periventricular foci of white matter demyelination with slight bifrontal predominance. CT of the brain revealed no abnormalities.

 

Along with the imaging studies, basic complete blood count (CBC), complete metabolic profile (CMP), urinalysis, cerebrospinal fluid (CSF) analysis, serum immunochemistry and serology, and infectious disease workup were ordered to determine possible etiologies of the patient's presenting symptoms that correlate with the imaging findings. The patient's initial CBC was unremarkable aside from an elevated neutrophil count of 11,000, and the urinalysis also returned normal. However, the CMP revealed numerous abnormalities. Potassium levels were measured at 3.1 mmol/L, glucose at 135 mmol/L, creatinine at 0.5 mg/dL, albumin at 5.1 g/dL, globulin at 3 g/dL, and the blood urea nitrogen (BUN)/creatinine ratio at 24 mg/dL (Table 1).

**Table 1:** Complete metabolic profile on March 25, 2021. A/G, albumin-globulin; AST, aspartate aminotransferase; ALT, alanine transaminase; AGAP, anion gap; BUN, blood urea nitrogen; CPK, creatine phosphokinase; SGPT, serum glutamic-pyruvic transaminase

Complete metabolic profile		
Test	Results	Reference
Sodium	139 mmol/L	136-144 mmol/L
Potassium	3.1 mmol/L (Low)	3.7-5.1 mmol/L
Chloride	98 mmol/L	96-106 mmol/L
CO_2_	30 mmol/L (High)	23-29 mmol/L
Glucose	135 mg/dL (High)	77-99 mg/dL
BUN	12 mg/dL	6-24 mg/dL
Creatinine	0.5 mg/dL (Low)	0.74-1.35 mg/dL
Calcium	9.7 mg/dL	8.5-10.2 mg/dL
Total protein	8 g/dL	6-8.3 g/dL
Albumin	5.1 g/dL (High)	3.5-5 g/dL
Total bilirubin	0.5 mg/dL	0.1-1.2 mg/dL
Alkaline phosphatase	78 IU/L	44-147 IU/L
AST	26 U/L	8-33 U/L
ALT (SGPT)	23 U/L	4-36 U/L
BUN/creatinine ratio	24 (High)	10:1 to 20:1
Globulin	3 g/dL	2-2.9 g/dL
A/G ratio	2	
AGAP	11 mEq/L	4-12 mEq/L
Total CPK	114 mcg/L	10-120 mcg/L

Concurrent with obtaining and evaluating the CBC and CMP, an infectious disease panel was also carried out to identify the possible virus and bacterial infection (Table 2). An autoimmune panel (Table 3) was also carried out to ensure no other autoimmune etiology. However, all results of that panel came back negative.

**Table 2:** Infectious disease panel. Ab, antibody; CMV, cytomegalovirus; EBV, Epstein-Barr virus; VDRL, Venereal Disease Research Laboratory; IgG, immunoglobulin G; IgM, immunoglobulin M; PCR, polymerase chain reaction; VZV, varicella-zoster virus

Infectious disease workup		
Test	Results	Reference
Syphilis serology (VDRL)	Nonreactive	Nonreactive
Lyme total Ab	0.29	<0.90 (negative)
Chikungunya virus IgG	0.18	0.79 or less
Chikungunya virus IgM	0.58	0.79 or less
SARS-CoV-2 PCR	Positive	Not detected
CMV DNA quantification Ab	Negative	Not detected
CMV DNA PCR log10	Test not performed	Not detected
CMV IgG index	5.4	<0.5 U/mL
Enterovirus RNA PCR	Negative	Not detected
EBV IgM Ab titer	<0.2	<18 U/mL
EBV capsid Ag IgG Ab	>0.8	<18 U/mL
EBV nuclear Ag Ab	>8.0	<18 U/mL
Herpes Simplex 1 IgG index	1.6	<0.8 U/mL
Herpesvirus 6 IgG Ab	0.26	Below limit of detection
Herpesvirus 6 IgM Ab	<1:20	<1:10 (Below limit of detection)
HIV 1 and HIV 2 Ag/Ab	Nonreactive	Not detected
Influenza A rapid	Negative	Not detected
Influenza B rapid	Negative	Not detected
Mumps virus IgM Ab	<0.80	0.79 or less
Rubeola IgG	1.2	13.4 or less
Rubeola IgM	<0.91	<1.20
VZV IgG Ab	4.8	134.9 or less
VZV IgM Ab	<0.91	<0.91

**Table 3:** Autoimmune panel. Ab, antibody; ANA, antinuclear antibody; RNP, ribonucleoprotein; GPI, glycoprotein I

Autoimmune panel		
Test	Result	Reference
Rheumatoid factor	Negative	Normal: negative
ANA screen	Negative	Normal: negative
Jo-1	<0.2	<1
SS-A/Ro Ab	<0.2	<1
SS-B/La Ab	<0.2	<1
Smith Ab	<0.2	19 or less
RNP Ab	<0.2	19 or less
Scl-70 Scleroderma Ab	<0.2	<1
Double-stranded DNA Ab	1.1	<4.9
MAG IgM Ab	<800	<1:1,600
Beta-2-GPI IgG Ab	<2.4	<15
B Beta-2-GPI IgM Ab	1.4	<15
Thyroglobulin Ab	<20	<20
Thyroid peroxidase Ab	<10	<35
Cardiolipin IgG Ab	<1.4	<40
Cardiolipin IgM Ab	1.4	<40
Complement C3	127	80-178
Complement C4	33.7	12-42

A serological study was also conducted to see if any immunological markers or bacterial/viral antigens could be detected on PCR. However, similar to the aforementioned autoimmune panel, all values that were tested for came back negative (Table 4).

**Table 4:** Immunology/serology panel (March 27, 2021). CSF, cerebrospinal fluid; RSV, respiratory syncytial virus; PCR, polymerase chain reaction; MRSA, methicillin-resistant Staphylococcus aureus

Immunology/Serology		
Test	Result	Reference
SARS-CoV-2 COVID-19 PCR	Positive	Not detected
SARS-CoV-2 COVID-19 IgG	Positive	Not detected
Adenovirus PCR	Negative	Not detected
Antinuclear antibody	Negative	Not detected
SSA IgG	Negative	Not detected
SSB IgG	Negative	Not detected
Bordetella pertussis PCR	Not detected	Not detected
Bordetella parapertussis PCR	Not detected	Not detected
Chlamydia pneumonia PCR (not COVID-19)	Not detected	Not detected
Coronavirus 229E PCR (not COVID-19)	Not detected	Not detected
Coronavirus HKU1 PCR (not COVID-19)	Not detected	Not detected
Coronavirus NL63 PCR (not COVID-19)	Not detected	Not detected
Coronavirus DC43 PCR (not COVID-19)	Not detected	Not detected
Influenza B PCR	Not detected	Not detected
Influenza A PCR	Not detected	Not detected
Metapneumovirus PCR	Not detected	Not detected
Mycoplasma pneumoniae PCR	Not detected	Not detected
Myelin basic protein CSF	>128 ng/mL (High)	<4 ng/mL
RSV PCR	Not detected	Not detected
MRSA rapid screen	Not detected	Not detected
Rhinovirus PCR	Not detected	Not detected
Parainfluenza 1 PCR	Not detected	Not detected
Parainfluenza 2 PCR	Not detected	Not detected
Parainfluenza 3 PCR	Not detected	Not detected
Parainfluenza 4 PCR	Not detected	Not detected

Finally, the CSF was checked for particular markers that could indicate that a viral pathogen was responsible for the patient's transverse myelitis (Table 5). Upon evaluation of the patient's CSF, it was noted that the total protein level was elevated at 87 mg/dL, glucose was within the normal range at 63 mg/mL, and the IgG level was elevated at 9.7. In addition to the parameters observed, the level of myelin basic protein observed in the CSF was above 128 ng/mL. According to the most recently accepted guidelines, a value between 4 and 9 ng/mL indicates a long-term breakdown of myelin basic protein. In contrast, a level >9 ng/mL is most associated with an active breakdown of myelin at the time of CSF extraction. Due to the patient's myelin basic protein level being more than 14× the accepted cutoff value, it was determined that the patient is undergoing active destruction of myelin. All these laboratory values, when put together, indicate that a viral etiology is the cause of this patient's transverse myelitis.

**Table 5:** CSF evaluation (March 26, 2021). CSF, cerebrospinal fluid; IgG, immunoglobulin G; PCR, polymerase chain reaction

CSF evaluation	Measured	Reference
Glucose	63 mg/dL	50-75 mg/dL
IgG	9.7 (High)	0-0.7 mg/L
Total protein	87 (High)	20-40 mg/dL
Clarity	Hazy	N/A
Color	Light pink	Clear
Neutrophils	58	<5 per mm³
Lymphocytes	20	<5 per mm³
Monocytes	22	<5 per mm³
Differential	Yes	N/A
RBC	590 (High)	0-10 per mm³
Tube# CSF	1;4	N/A
WBC	C124, C169	0-10 per mm³
Albumin	54 (High)	8-37 mg/dL
IgG synthesis rate	12.5 (High)	<12 mg/24 hours
IgG/albumin ratio	0.18	0.066-0.270
Oligoclonal bands	Negative	Negative
Oligoclonal bands number	0	0
Meningitis encephalitis specimen type	CSF	CSF
Escherichia coli K1 PCR	Negative	Negative
Hemophilus influenzae PCR	Negative	Negative
Listeria monocytogenes PCR	Negative	Negative
Neisseria meningitidis PCR	Negative	Negative
Streptococcus group B PCR	Negative	Negative
Streptococcus pneumoniae PCR	Negative	Negative
Cytomegalovirus CSF qualitative PCR	Negative	Negative
Enterovirus RNA PCR	Negative	Negative
Epstein-Barr virus RNA PCR	Negative	Negative
Herpes virus 6 CSF qualitative PCR	Negative	Negative
Herpes simplex 1 PCR	Negative	Negative
Herpes simplex 2 PCR	Negative	Negative
Human parechovirus PCR	Negative	Negative
Varicella zoster PCR	Negative	Negative
Cryptococcus neoformans gattii PCR	Negative	Negative

Upon receiving the results of the ordered laboratory tests and imaging studies, the patient was treated with intravenous (IV) steroids, plasmapheresis, and rituximab. Initiation of treatment began with the administration of high-dose IV methylprednisolone. The patient was administered 1,000 mg/day of methylprednisolone for 3 days (total 3,000 mg; maximum suggested dosage) over four treatments per day for one hour each at a rate of 250 mg/hour every six hours. This was carried out because steroids are well proven to be a first-line agent to reduce generalized inflammation. Another treatment this patient underwent was plasmapheresis, which was performed daily during the entire hospital stay to remove any antibodies or proteins that were eliciting this immune response leading to her condition. Finally, the monoclonal antibody rituximab was given to alleviate the immune response in the patient further. As per standard protocols, Rituxan (rituximab) was administered as two 500 mg IV infusions separated by two weeks. The medication is widely known to inhibit T-helper cells, B-cells, and macrophages and induce the activity of T-regulatory cells, which help suppress the immune response.

Through the effects of the medications and treatment modalities, the patient's functioning was partially restored. The patient was able to ambulate appropriately, albeit with short distances. The episodes of incontinence completely ceased, and all paresthesia was significantly diminished. The MRI of the thoracic spine revealed a T2 signal of the spinal cord that was less intense than previously recorded. Imaging two weeks after treatment also showed a resolution of hyperintensity that was apparent on the MRI before treatment. However, the patient was advised to follow up as an outpatient with the neurologist and primary care physician five to seven days after hospital discharge. The follow-up was advised to confirm that symptoms did not return and to undergo further imaging later to ensure that positive changes were not due to ongoing plasmapheresis.

## Discussion

Due to the short- and long-term neurological complications associated with COVID-19, it is of the utmost clinical significance to identify and treat potential neurological complications caused by the virus. Neurological manifestations may range from a simple headache to life-threatening encephalitis. Healthcare providers must be aware of such complications as they may be the initial presenting sign or a late manifestation of COVID-19 infection. In this case, we presented an elderly adult woman who developed TM with T2 weighted hyperintensity on the cervical to the lumbar spine. The patient's CSF analysis was consistent with laboratory findings that would be associated with viral infection of the central nervous system. While the infectious disease panel was negative for all other pathogens tested (Table 2), due to the temporal relationship between COVID-19 infection in this patient and the onset of neurological symptoms, we propose that the robust immune response produced in response to COVID-19 infection led to the neurological symptoms experienced in this patient. This pathogenesis is similar to how TM is caused in patients afflicted with more classic pathogens associated with the disease, such as the Epstein-Barr virus (EBV) and herpes simplex virus (HSV) [11].

TM is defined as a rare inflammation of the spinal cord, which leads to numerous neurological deficits. Symptoms include but are not limited to sensory loss, rapid onset of weakness in the extremities, and bladder or bowel dysfunction. Inflammation may occur at any spinal cord level, leading to various symptoms that vary among patients. Most cases of TM are idiopathic with no predisposing factors; however, some infectious agents may also cause TM. Numerous viral agents, including the varicella-zoster virus, HIV, human T-lymphotropic virus 1 (HTLV-1), West Nile virus, and Zika virus, have been documented as causing TM. COVID-19 may be another viral etiology leading to TM in infected individuals. Mycoplasma and Treponema pallidum have also been known to cause TM. Numerous autoimmune disorders have been known to cause TM, such as Sjogren's syndrome, sarcoidosis, systemic lupus erythematosus, and rheumatoid arthritis. In the United States alone, there are approximately 1,400 cases of TM reported each year. The age of affected individuals varies, with the most common ages being 10-19 and 30-39 years [11].

After careful consideration and thorough evaluation of this case, we postulate that the TM experienced by this patient may be related to her SARS-CoV-2 inoculation. However, the patient's serology did test positive for CMV, HSV1, and EBV, three pathogens that have a known association with TM onset. In this case, these viruses were ruled out as the causative agents as the patient's CSF returned negative for all three pathogens, leading us to conclude that SARS-CoV-2 may be the causative agent. Two potential theories describe how SARS-CoV-2 may cause neurological complications such as TM. The first is through direct neuroinvasion in which SARS-CoV-2 infects the brain and spinal cord through its binding receptor, angiotensin-converting enzyme 2 (ACE2) [3]. ACE2 receptors are most abundantly found in the lung cells, leading to the upper respiratory manifestations most often seen in COVID-19-positive individuals. A literature review by Lewis et al. [12] identified 321 patients who presented with neurological complications while being infected by COVID-19. Those 321,304 patients' CSF was tested for SARS-CoV-2 RNA using PCR. Seventeen patients' CSF results returned positive for SARS-CoV-2 RNA. While unlikely, the presence of SARS-CoV-2 in the CSF may be evidence of the virus's ability to cause direct neuroinvasion, which produces neurological complications in infected patients.

Furthermore, the second theory postulates that neurological manifestations caused by COVID-19 are due to an exaggerated immune response causing the spinal cord inflammation seen in acute TM [3]. The increased immune response leads to demyelination in acute TM cases. While the patient's CSF was not tested for COVID-19 using PCR, we believe that some combination of both theories of direct neuroinvasion and systemic inflammation may have led to the development of acute TM in this patient.

## Conclusions

As more information becomes available regarding the ongoing COVID-19 pandemic, special notes should be taken of those patients who present with nonspecific and specific neurological complications. TM can be a potentially devastating disease. If a causal relationship between acute TM and COVID-19 is established, healthcare providers should be aware of the potential implications. COVID-19 should be considered one of the many etiologies that may lead to the development of acute TM within the currently available literature. Further research should be conducted into the potential ability of SARS-CoV-2 to cause neurological damage. While not all evidence is yet available, this case should provide proof of yet another complexity due to COVID-19.

## References

[1] Padda I, Parmar M (2022). COVID (SARS-COV-2) Vaccine. In: StatPearls.

[2] Makhoul E, Aklinski JL, Miller J (2022). A review of COVID-19 in relation to metabolic syndrome: obesity, hypertension, diabetes, and dyslipidemia. Cureus.

[3] Padda I, Khehra N, Jaferi U, Parmar MS (2020). The neurological complexities and prognosis of COVID-19. SN Compr Clin Med.

[4] Pennisi M, Lanza G, Falzone L, Fisicaro F, Ferri R, Bella R (2020). SARS-CoV-2 and the nervous system: from clinical features to molecular mechanisms. Int J Mol Sci.

[5] Advani S, Hosseini SM, Zali A, Ommi D, Fatemi A, Khoshnoud RJ, Ashrafi F (2021). Transverse myelitis after SARS-CoV-2 infection: report of two cases with COVID-19. Clin Case Rep.

[6] Kara S, Candelore T, Youssef P, Nedd K (2021). Evidence of post-COVID-19 transverse myelitis demyelination. Cureus.

[7] Sarma D, Bilello LA (2020). A case report of acute transverse myelitis following novel coronavirus infection. Clin Pract Cases Emerg Med.

[8] Durrani M, Kucharski K, Smith Z, Fien S (2020). Acute transverse myelitis secondary to severe acute respiratory syndrome coronavirus 2 (SARS-CoV-2): a case report. Clin Pract Cases Emerg Med.

[9] Chow CC, Magnussen J, Ip J, Su Y (2020). Acute transverse myelitis in COVID-19 infection. BMJ Case Rep.

[10] Arslan D, Acar-Ozen P, Gocmen R, Elibol B, Karabudak R, Tuncer A (2022). Post-COVID-19 longitudinally extensive transverse myelitis: is it a new entity?. Neurol Sci.

[11] Simone CG, Emmady PD (2022). Transverse Myelitis. In: StatPearls.

[12] Lewis A, Frontera J, Placantonakis DG, Galetta S, Balcer L, Melmed KR (2021). Cerebrospinal fluid from COVID-19 patients with olfactory/gustatory dysfunction: a review. Clin Neurol Neurosurg.

